# Comparison of the dosimetry of scandium-43 and scandium-44 patient organ doses in relation to commonly used gallium-68 for imaging neuroendocrine tumours

**DOI:** 10.1186/s40658-024-00669-5

**Published:** 2024-07-15

**Authors:** Carlos Vinícius Gomes, Bruno Melo Mendes, Lucas Paixão, Silvano Gnesin, Cristina Müller, Nicholas P. van der Meulen, Klaus Strobel, Telma Cristina Ferreira Fonseca, Thiago Viana Miranda Lima

**Affiliations:** 1 Graduate School for Cellular and Biomedical Sciences, University of Bern, 3012 Bern, Switzerland; 2 Post-graduation Program in Nuclear Sciences and Techniques, Department of Nuclear Engineering, Federal University of Minas Gerais, Belo Horizonte, Brazil; 3 Nuclear Technology Development Center – CDTN/CNEN, Belo Horizonte, Brazil; 4 Department of Anatomy and Imaging, Federal University of Minas Gerais, Belo Horizonte, Brazil; 5 Institute of Radiation Physics, Lausanne University Hospital, University of Lausanne, Lausanne, Switzerland; 6 Institute of Radiology and Nuclear Medicine, Luzerner Kantonsspital – LUKS, Lucerne, Switzerland; 7 Center for Radiopharmaceutical Sciences, Paul Scherrer Institute (PSI), Villigen, Switzerland; 8 Laboratory of Radiochemistry, Paul Scherrer Institute (PSI), Villigen, Switzerland; 9 Faculty of Health Sciences and Medicine, University of Lucerne, Lucerne, Switzerland

**Keywords:** Radiopeptides, Scandium, Dosimetry, Monte Carlo, Neuroendocrine tumours, PET

## Abstract

**Background:**

Several research groups have explored the potential of scandium radionuclides for theragnostic applications due to their longer half-lives and equal or similar coordination chemistry between their diagnostic and therapeutic counterparts, as well as lutetium-177 and terbium-161, respectively. Unlike the gallium-68/lutetium-177 pair, which may show different in-vivo uptake patterns, the use of scandium radioisotopes promises consistent behaviour between diagnostic and therapeutic radiopeptides. An advantage of scandium’s longer half-life over gallium-68 is the ability to study radiopeptide uptake over extended periods and its suitability for centralized production and distribution. However, concerns arise from scandium-44’s decay characteristics and scandium-43’s high production costs. This study aimed to evaluate the dosimetric implications of using scandium radioisotopes with somatostatin analogues against gallium-68 for PET imaging of neuroendocrine tumours.

**Methods:**

Absorbed dose per injected activity (AD/IA) from the generated time-integrated activity curve (TIAC) were estimated using the radiopeptides [^43/44/44m^Sc]Sc- and [^68^Ga]Ga-DOTATATE. The kidneys, liver, spleen, and red bone marrow (RBM) were selected for dose estimation studies. The EGSnrc and MCNP6.1 Monte Carlo (MC) codes were used with female (AF) and male (AM) ICRP phantoms. The results were compared to Olinda/EXM software, and the effective dose concentrations assessed, varying composition between the scandium radioisotopes.

**Results:**

Our findings showed good agreement between the MC codes, with − 3 ± 8% mean difference. Kidneys, liver, and spleen showed differences between the MC codes (min and max) in a range of − 4% to 8%. This was observed for both phantoms for all radiopeptides used in the study. Compared to Olinda/EXM the largest observed difference was for the RBM, of 21% for the AF and 16% for the AM for scandium- and gallium-based radiopeptides. Despite the differences, our findings showed a higher absorbed dose on [^43/44^Sc]Sc-DOTATATE compared to its ^68^Ga-based counterpart.

**Conclusion:**

This study found that [^43/44^Sc]Sc-DOTATATE delivers a higher absorbed dose to organs at risk compared to [^68^Ga]Ga-DOTATATE, assuming equal distribution. This is due to the longer half-life of scandium radioisotopes compared to gallium-68. However, calculated doses are within acceptable ranges, making scandium radioisotopes a feasible replacement for gallium-68 in PET imaging, potentially offering enhanced diagnostic potential with later timepoint imaging.

**Supplementary Information:**

The online version contains supplementary material available at 10.1186/s40658-024-00669-5.

## Introduction

The suitability of scandium radionuclides for theragnostic applications has been proposed and evaluated by several research groups [1–4]. The rationale for using positron-emitting scandium radioisotopes (scandium-43 and scandium-44) is to benefit both from their longer half-lives (t_1/2_ = 3.89 h and 4.04 h, for scandium-43 and scandium-44, respectively) and identical coordination chemistry to its therapeutic counterpart scandium-47 (t_1/2_ = 3.35 days) [5, 6]. Such specificity of scandium radioisotopes might improve the theragnostic approach as compared to the currently-used theragnostic couple gallium-68/lutetium-177 that, due to the different coordination chemistry, may result in a non-negligible variability of the in-vivo uptake patterns between diagnostic and therapeutic radiopeptides [4, 6, 7]. A major advantage of employing scandium-43 and scandium-44 resides in the possibility to explore the radiopeptide biokinetic for an extended time interval (within 12 h post administration), relevant to provide more accurate therapeutic extrapolation. This feature is limited when using gallium-68 (t_1/2_ = 68 min) in relation with lutetium-177 (t_1/2_ = 6.64 days) or terbium-161 (t_1/2_ = 6.95 days) radiolabelled compounds. Moreover, the extended half-lives of scandium radioisotopes would facilitate a centralized production of scandium-based radiopeptides and their distribution over larger distances from the production site [8]. A potential drawback of using scandium-43/scandium-44 might consist of an increased absorbed dose in organ-at-risk (OAR), which is expected if there is a longer half-life from scandium, but similar biological half-life compared with gallium-68. Additionally, scandium-44, due to its non-pure positron decay associated with high-energy prompt gamma emission at 1157 keV (99.8%), would require improved radiation protection procedures [9]. However, another potential PET nuclide is the scandium-44m (t_1/2_ = 58.7 h), which has a 1.2% positron decay to calcium-44 and a 98.8% decay to scandium-44 [10]. Due to its relatively long half-life, it might be a relevant factor for the dose estimation compared to scandium-43/44.

Our prior research [11] demonstrated that, in terms of instrumentation, current clinical positron emission tomography (PET) exhibited comparable performance using scandium-44 or a mixture of scandium-43/scandium-44 with ones achieved with fluorine-18 or gallium-68 PET. The practical drawback of scandium-43 is associated with its high costs for production, which rise considerably when high purity is required [8, 12, 13].

The objective of this study was to assess the dosimetric impact of scandium radioisotopes (at varying levels of radionuclide purity), used in tandem with somatostatin analogues, compared to gallium-68 for PET of neuroendocrine tumour (NET) imaging.

## Material and methods

In this study, we estimated organ absorbed dose (AD) and the subject effective doses for the following PET radionuclides: scandium-43, scandium-44, scandium-44m, and gallium-68, all conjugated with DOTATATE, utilizing the Olinda/EXM (Organ Level Internal Dose Assessment Code—Exponential Modelling) software version 2.1.1 [14]. For simplicity, the radiopeptides [^43/44/44m^Sc]Sc-DOTATATE and [^68^Ga]Ga-DOTATATE will henceforth be referred to as scandium-43/44/44m and gallium-68, respectively. Olinda/EXM is a well-characterized, robust software used for internal dosimetry assessment based on the MIRD formalism. In addition, we employed the Electron Gamma Shower (EGSnrc) [15] and Monte Carlo N-Particle (MCNP6.1) [16] as Monte Carlo (MC) codes for dosimetry computations. Employing two different MC codes allowed cross-verification and validation of results.

Assuming as known $TAC_{SR,Lu - 177}$ (t), the time activity curve (TAC) in a given source region (SR) of a patient administered with a ^177^Lu-labeled radiopharmaceutical, the TAC for a radiolabeled compound having the same molecular vector and a different PET radionuclide: $TAC_{SR,PR}$ (t), for which we can assume same biological behavior, can be obtained in a few steps. First, we remove the physical decay component of the lutetium-177 from $TAC_{SR,Lu - 177}$ (t), hence, obtaining a TAC that accounts only for the biological component characteristic of the molecular probe used (in our case, the DOTATATE molecule):

1 $$TAC_{SR,biol} \left( t \right) = TAC_{SR,Lu - 177} \left( t \right) \times e^{{\frac{{{\text{ln}}\left( 2 \right) \times t}}{{T_{phys,Lu - 177} }}}}$$

where $T_{phys,Lu - 177}$ is the physical half-life to lutetium-177. It is worth noting that in the present study, $TAC_{SR,biol} \left( t \right)$ is assumed to be independent from the radionuclide used to label the molecular probe. In a second step, we apply the PET-specific radionuclide physical decay component: $e^{{\frac{{ - {\text{ln}}\left( 2 \right) \times t}}{{T_{phys,PR} }}}}$ to $TAC_{RS,biol}$ to obtain:

2 $$TAC_{SR,PR} \left( t \right) = TAC_{SR,biol} \left( t \right) \times e^{{\frac{{ - {\text{ln}}\left( 2 \right) \times t}}{{T_{phys,PR} }}}} .$$

The time integrated activity coefficient (TIAC) for a specific SR and PET radionuclide combination is then obtained by time integration of the normalised TAC (i.e.: nTAC = TAC/A_admin_) as follows:

3 $$TIAC_{SR,PR} = \mathop \smallint \limits_{0}^{\infty } nTAC_{SR,PR} \left( t \right)dt = \mathop \smallint \limits_{0}^{\infty } nTAC_{SR,biol} \left( t \right) \times e^{{\frac{{ - {\text{ln}}\left( 2 \right) \times t}}{{T_{phys,PR} }}}} dt.$$

Using Eq. 1 in Eq. 3, the propriety of linearity of the integral operator and the known analytical solution for the integral of the exponential function (i.e.: $\mathop \smallint \limits_{0}^{\infty } e^{ - \alpha t} dt = 1/\alpha$), we then obtain:

4 $$TIAC_{SR,PR} = TIAC_{SR,Lu - 177} \times \frac{{T_{phys,SR,PR} }}{{T_{phys,SR,Lu - 177} }}.$$

In this study, we estimated TIAC values for SR: spleen, red bone marrow (RBM), liver, kidneys, and the rest of the body (hereon referred to as “remainder”), using $TIAC_{SR,Lu - 177}$ reported in the publication of Marin et al. [17].

Source organ TIACs for the radioisotopes concerned, are reported in Table 1.

**Table 1:** Estimated source organ TIAC (MBq-h/MBq) for lutetium-177, scandium radioisotopes (scandium-43/44/44m), and gallium-68

Organ	Lutetium-177	Scandium-44	Scandium-44m	Scandium-43	Gallium-68
Right kidney	1.6000	0.0406	0.6000	0.0405	0.0120
Left kidney	1.6000	0.0406	0.6000	0.0405	0.0120
Spleen	2.5000	0.0635	0.9375	0.0633	0.0180
Liver	15.2000	0.3859	5.7000	0.3849	0.1120
RBM	0.3000	0.0076	0.1125	0.0076	0.0020
Reminder	12.5000	0.3174	4.6875	0.3166	0.0920

**Fig. 1:**
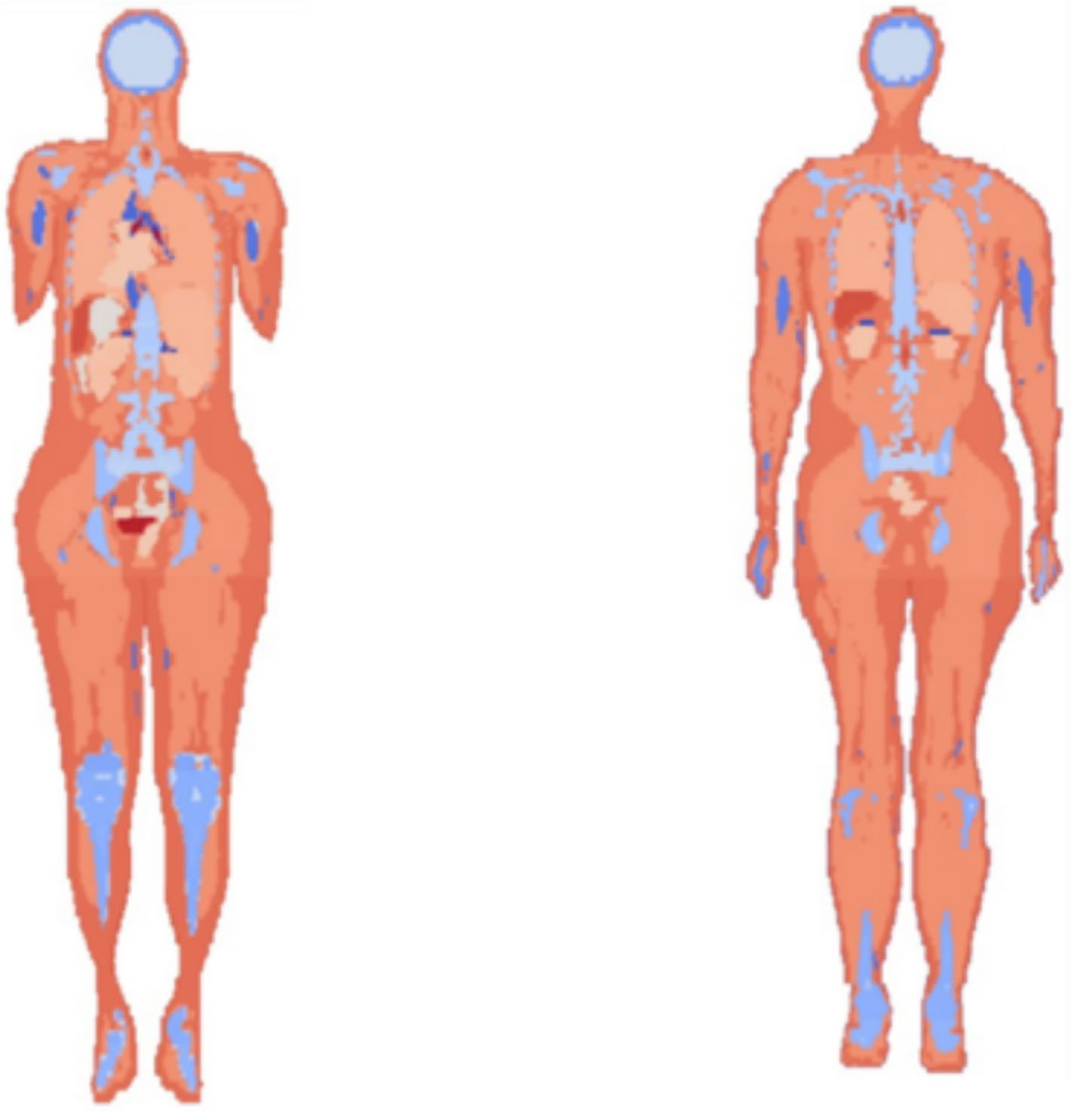
Computational phantoms of the reference ICRP 110: female (AF), left, and male (AM), right

Computational phantoms geometries can be integrated in the MC simulations to simulate the transport of radiation through matter. The adult human ICRP 110 reference phantoms, female (AF) and male (AM), shown in Fig. 1, were adopted for this study [18].

*EGSnrc* was used for simulating the radiation transport and collecting the absorbed dose (AD) (Gy) for each region/tissue of the body. The *egs_source_collection* and *egs_dose_scoring* libraries were used to implement the decay distribution according to TIAC previously estimated for the scandium isotopes and to score the AD in the target tissues of the ICRP 110 adult human phantom (Fig. 1). A number of 2 × 10^8^ initial particles were scored in each simulation in order to keep the statistical uncertainties below 5%. In this work, the deposited energy in tissues was obtained employing the *tutor7pp C*++ application as part of the EGSnrc MC toolkit.

*MCNP6.1* was used for scoring ADs within voxel grids of the reference adult phantoms. The densities and chemical compositions of the organs and tissues represented in the simulations were based on the publication of ICRP 110 [18]. The TIAC used to simulate the decay distribution in the organs of the phantoms was based on Table 1. Emissions in source organs were implemented in accordance with the recommendations of ICRP 133 [19]. A sampling algorithm, weighted by tissue masses, was used for distribution of decays in source regions composed of two or more different tissues. The DECDATA® software [20] provides energy data and respective probability for scandium isotopes decays. A summary of scandium-43/44/44m, lutetium-177, and gallium-68 decay data is presented in Table 2. Detailed decay data used in this study can be accessed through the software DECDATA® software available on the ICRP website. Tally + F6, which returns absorbed dose (MeV g^−1^), was used in dosimetry of the tissues and organs segmented in the phantoms. The simulations involved an average of 5E+08 initial particles (NPS), ensuring robust statistical significance. Each simulation run lasted approximately 8 h per input file, running in a single core of an Intel i7 processor.

**Table 2:** Summary of the decay data for the radionuclides evaluated in this study

Radiation type	Scandium-43		Scandium-44		Scandium-44m		Lutetium-177		Gallium-68	
	Yi	Ei	Yi	Ei	Yi	Ei	Yi	Ei	Yi	Ei
γ rays	2.3E−01	3.7E−01	1.0E+00	1.2E+00	9.0E−01	3.0E−01	1.8E−01	1.8E−01	3.6E−02	1.1E+00
X rays	4.6E−01	1.7E−04	2.2E−01	1.7E−04	9.9E−01	l.0E−04	1.4E+0D	2.6E−03	5.7E−01	7.2E−04
AP	1.8E+00	5.1E−01	1.9E+00	5.1E−01	–	–	–	–	1.8E+00	5.1E−01
*β* +	8.8E−01	4.8E−01	9.4E−01	6.3E−01	–	–	–	–	8.9E−01	8.3E−01
*β* −	–	–	–	–	–	–	1.0E+00	1.3E−01	–	–
ICE	1.3E−04	3.7E−01	6.5E−05	1.2E+00	1.2E−01	2.7E−01	1.5E−01	8.7E−02	9.3E−06	1.1E+00
AE	3.2E−01	1.1E−03	1.5E−01	1.1E−03	8.3E−01	S.3E−04	1.1E+00	1.0E−03	4.1E−01	1.3E−03

Yi, Intensity of radiation i per decay; Ei, Energy of radiation i in MeV; AP, Annihilation photons; ICE, Internal conversion electrons; AE, Auger electrons

MC codes calculate the absorbed dose in a targeted-organ per emitted particle in source-organ. The absorbed dose per injected activity (mGy/MBq) was calculated as the product of the absorbed dose per emitted particle; the source organ time-integrated activity coefficient of the respective scandium isotope; and the number of particles emitted per decay for a determined scandium isotope, provided by ICRP 107 [20]. Details about this method can be observed in the supplementary file.

### Analysis

Organ ADs, effective doses (E), and their standard deviations, were obtained using two computational methods: OLINDA/EXM 2.1 and MC codes. In addition, organ AD were estimated for a mixture with varying concentrations of the scandium radionuclides, as shown in Table 3, by varying concentrations of scandium-44 (from 0 to 100%, in 10% increments), scandium-44m (from 0% of the scandium-44 concentration to 15%, in steps of 1%), and scandium-43 (from 0 to 100%, in steps of 10%).

**Table 3:** Varying composition of the radionuclide texture considered in AD calculations

Radionuclide	Composition										
Scandium-44	100%	90%	80%	70%	60%	50%	40%	30%	20%	10%	0%
Scandium-44m	0% of the scandium-44	0%	0%	0%	···	···	···	···	···	···	0%
	1% of the scandium-44	1%	1%	1%	···	···	···	···	···	···	0%
	2% of the scandium-44	2%	2%	2%	···	···	···	···	···	···	0%
	···	···	···	···	···	···	···	···	···	···	0%
	15% of the scandium-44	15%	15%	15%	···	···	···	···	···	···	0%
Scandium-43	0%	10%	20%	30%	40%	50%	60%	70%	80%	90%	100%

The percentage differences ($\Delta$) were defined by the equation:

5 $$\Delta = \left( {1 - \frac{\delta }{\varepsilon }} \right)*100\% ,$$

where $\delta$ and $\varepsilon$ were combined as a ratio comparing EGSnrc vs MCNP, EGSnrc vs Olinda, and MCNP vs Olinda results.

## Results

S-values were derived using the MC method and followed the MIRD formalism. Initially, the absorbed dose per injected activity (AD/IA) for each region or tissue within the phantoms were computed.

Detailed AD/IA calculations for various organs, evaluated phantoms, and calculation methodologies (EGSnrc, MCNP6.1, and Olinda 2.1) are elaborated in the supplementary materials, as well as the mean values and relative differences (Eq. 5).

Comparative analysis of the AD/IA derived from each MC code revealed minimum and maximum disparities across the organs (kidneys, liver, spleen, and RBM) ranging from − 4 to 8%, averaging at − 3 ± 8%.

**Fig. 2:**
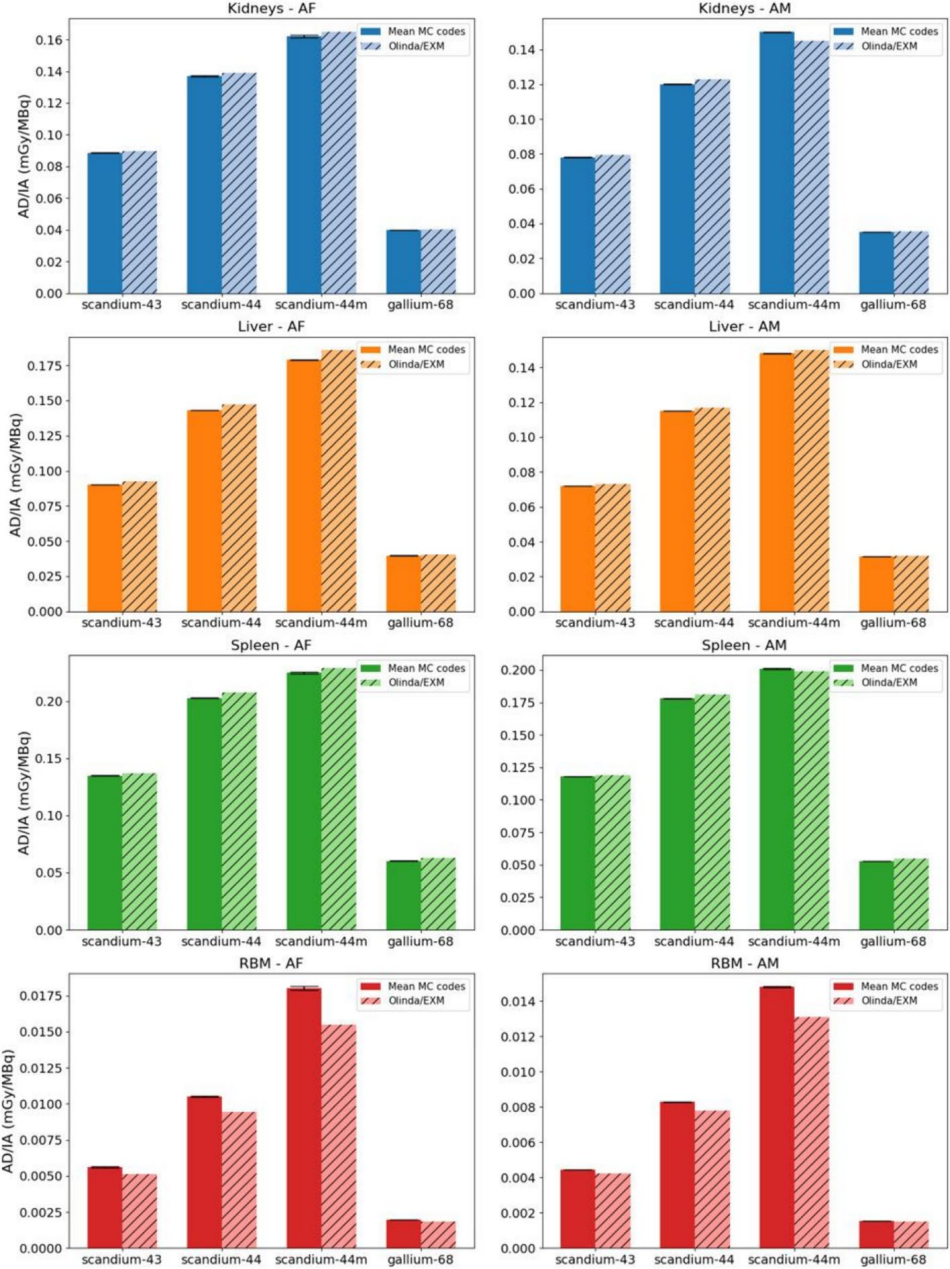
Absorbed dose per injected activity estimated from the mean MC codes and Olinda/EXM software by radionuclide. The results are represented for the female (AF) and male (AM) ICRP reference phantoms

Figure 2 offers a succinct representation of this data, showcasing results for the kidneys, liver, spleen, and RBM, segmented by phantoms. Minor or negligible variances were observed when juxtaposing the average AD/IA (Gy) outcomes of the MC codes against Olinda’s for the kidneys, liver, and spleen. A more significant contrast was noted for RBM AD/IA results, with the most pronounced discrepancy being 21% and 16% for the AF and AM, respectively, in the case of scandium-44m. The smallest differences stood at − 9% (AF) and − 4% (AM) for gallium-68. Error bars are depicted in Fig. 2; however, owing to the modest standard deviations, they might not be distinctly perceptible.

**Fig. 3:**
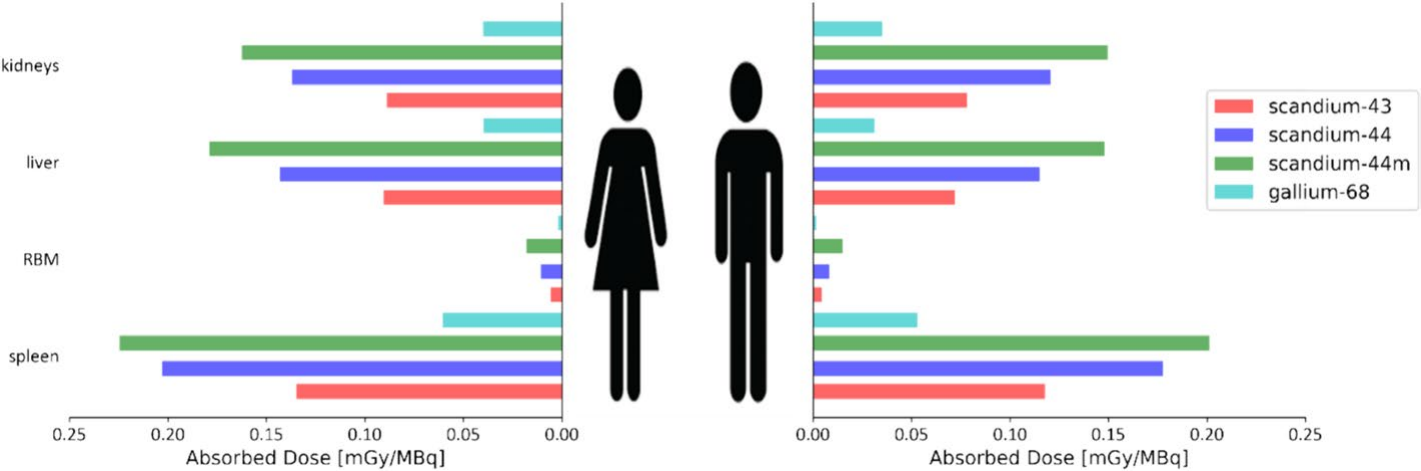
Organ dose comparison for both male and female phantoms for the different radioisotopes

An overview between mean absorbed doses in mGy (for the evaluated calculated method) per injected activity (in MBq) for the different radionuclides are presented in Fig. 3. Per MBq, the radionuclides with shorter half-lives translated into the lowest absorbed dose for all organs and effective dose (in mSv). The kidneys and spleen resulted in a higher AD/IA for the scandium-44 than scandium-44m. This systematic behaviour was observable for the MC codes and the Olinda results.

**Fig. 4:**
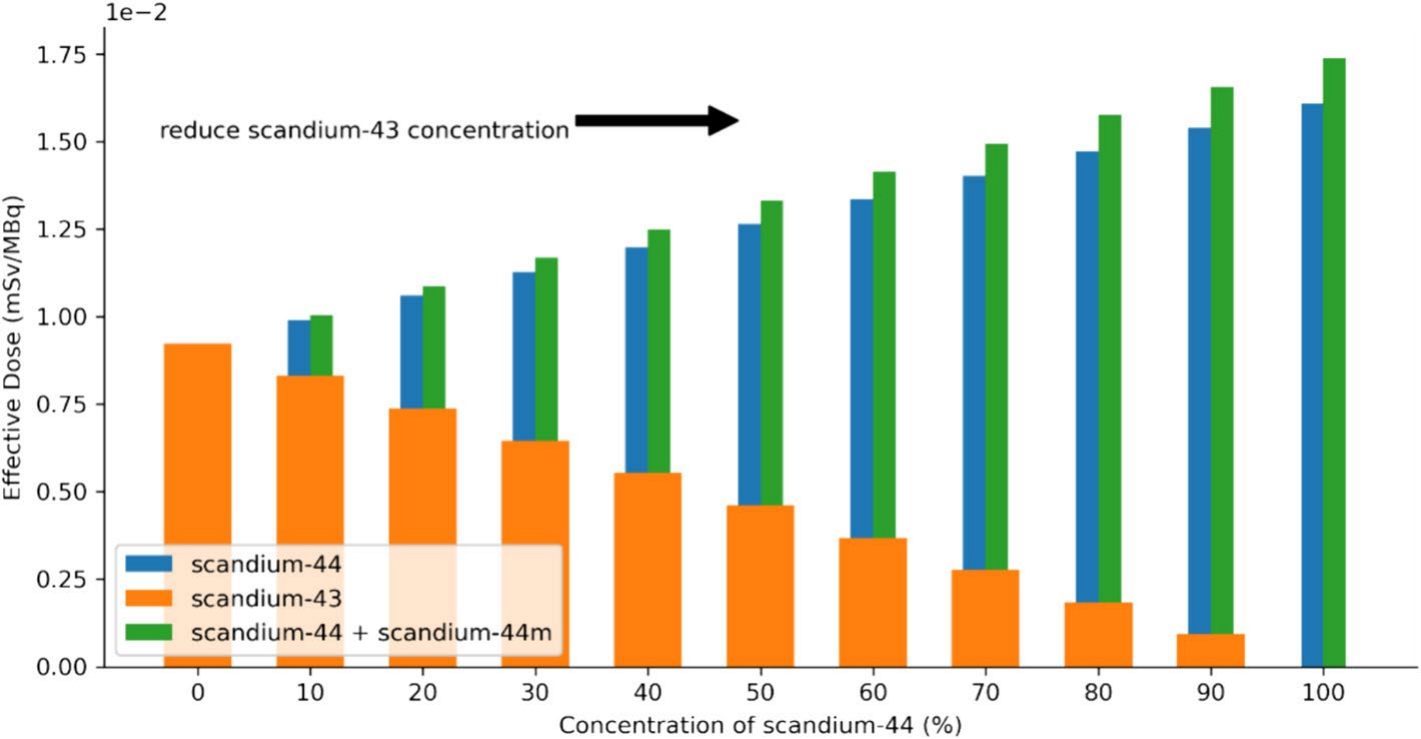
Effective dose comparison for the different scandium radioisotopes. Effect on scandium concentrations in the mixture between scandium-43, scandium-44, and scandium-44m

Additionally, Fig. 4 presents the mean effective doses per injected activity (mSv/MBq) for the different simulated scenarios of concentration between scandium-43, scandium-44, and scandium-44m. The results were evaluated from a mixture with varying concentrations of the scandium radionuclides as showed in Table 3, for both phantoms.

## Discussion

In this study, we evaluated the absorbed doses from different OAR and the effective dose for a range of scandium and gallium radionuclides. More specifically, this work investigates the dosimetry of DOTATATE labelled with variable radionuclides in view of their clinical application. In previous work, scandium-43 and scandium-44 were described as favourable radioisotopes for peptide-based PET imaging due to their physical characteristics [9, 11]. We also reported previously that the EGSnrc and MCNP6.1 MC codes have good agreement among the results when used for dosimetric purposes [21]. If both codes independently produce similar results, it increases confidence in the accuracy of the computed dosimetry. The only concern to the authors was the possible radiation exposure from these radioisotopes to the patient.

In DOTATATE peptide receptor radionuclide therapy (PRRT), the spleen, RBM, liver, and kidneys are known to retain the majority of the administered therapeutic activity and can potentially be associated to the development of radio-induced toxicity [22]. However, published dosimetry data are available only for [^68^Ga]Ga-DOTATATE [23–25].

In this study, the dosimetry data for the previously-mentioned diagnostic scandium radioisotopes were assessed and quantified. Comparable to the increased half-life, these radioisotopes translated into longer organ residence times. Furthermore, the scandium radioisotopes, scandium-43/44/44m, have similar characteristics to that of lanthanide therapeutic nuclides such as lutetium-177 and terbium-161. However, the dose quantification of scandium radioisotopes labelled to DOTATATE has not been available before this study.

Table 4 shows the main results for AD/IA for the OAR calculated in this work, including the dosimetry for the ^111^In-Octreotide, a somatostatin receptor also used for NETs [24]. Although somatostatin receptor imaging using PET has replaced scintigraphy imaging, [^111^In]In-Octreotide is still performed when PET is not available [26]. In these findings, the dosimetry for the scandium radioisotopes (scandium-43 and scandium-44) presented higher absorbed dose per injected activity compared to gallium-68 for the main OARs. The results are in accordance due to the half-life of these radioisotopes being considerably longer than gallium-68.

**Table 4:** Absorbed dose (mGy) and effective dose (mSv) per injected activity (MBq) for [^43/44/44m^Sc]Sc-DOTATATE, [^68^Ga]Ga-DOTATATE, and [^111^In]In-Octreotide

Phantom	Organs	DOTATATE				[^ln^In]In-Oetreotidc [21]
		Scandium-43	Scandium-44	Scandium-44m	Gallium-68	
AF	Kidneys	8.9E−2 ± 2.9E−4	1.4E−1 ± 3.4E−4	1.6E−1 ± 8.0E−4	4.0E−2 ± 9.4E−5	4.1E−1
AM		7.8E−2 ± 8.6E−5	1.2E−1 ± 1.3E−4	1.5E−1 ± 2.5E−4	3.5E−2 ± 7.7E−5	
	Liver	9.0E−2 ± 6.2E−5	1.4E−1 ± 8.4E−5	1.8E−1 ± 2.0E−5	4.0E−2 ± 2.2E−5	1.0E−1
		7.2E−2 ± 2.9E−5	1.2E−1 ± 4.4E−5	1.5E−1 ± 9.3E−5	3.1E−2 ± 1.7E−5	
	RBM	5.6E−3 ± 2.9E−5	1.0E−2 ± 5.0E−5	1.8E−2 ± 1.3E−5	2.0E−3 ± 8.2E−6	2.0E−2
		4.4E−3 ± 5.8E−6	8.3E−3 ± 1.2E−5	1.5E−2 ± 3.2E−5	1.5E−3 ± 5.5E−6	
	Spleen	1.3E−1 ± 2.8E−4	2.0E−1 ± 3.5E−4	2.2E−1 ± 7.0E−4	6.0E−2 ± 1.0E−4	5.7E−1
		1.2E−1 ± 1.4E−4	1.8E−1 ± 2.1E−4	2.0E−1 ± 3.3E−4	5.3E−2 ± 8.5E−5	
	Effective dose (mSv/MBq)	1.1E−2 ± 5.7E−6	2.0E−2 ± 9.6E−5	2.9E−2 ± 2.5E−5	4.5E−3 ± l.GE−5	5.4E−2
		9.2E−3 ± 1.2E−5	1.6E−2 ± 2.5E−5	2.5E−2 ± 6.8E−5	3.6E−3 ± 1.4E−5	

One of the physical characteristics of indium-111 is its long half-life (t_1/2_ = 2.8 days), quite similar compared to scandium-44m. Analysing the results, one can observe the same order of magnitude between them. On the other hand, the results obtained for the other scandium radioisotopes show good concordance when compared to [^111^In]In-Octreotide. In this case, the dose results for the [^43^Sc]Sc- and [^44^Sc]Sc-DOTATATE present delivering similar dose amount to the OARs in a short time compared to the well-established Octreotide.

Supplementary Table 8 presents the results of this work for [^68^Ga]Ga-DOTATATE. The highest organ absorbed doses were observed in the spleen, kidneys, liver, and gallbladder, in descending order, for both male and female phantoms. The male phantom mean spleen dose result from this work presented differences of 51%, 81%, and 79% when compared to the studies of Sandstrom et al. [23], Walker et al. [24], and Josefsson et al. [25], respectively. For the female phantom, the difference was 45% when comparing the spleen dose result of this work with the result from Sandstrom et al. [23]. A 79% and 76% difference for the spleen in the female phantom was found for Walker et al. [24] and Josefsson et al. [25], respectively. The absorbed dose to kidneys for the male phantom was within 62% of Walker et al. [24] and Sandstrom et al. [23] results, presenting a 75% difference regarding the Josefsson et al. [25] result. Liver dose differences ranged between 12 and 63%. Regarding effective dose coefficients, the results presented in this work (3.6E−3 and 4.5E−3 mSv/MBq for male and female phantoms, respectively) are quite different from the literature (> 65%). The studies involving patients report an effective dose coefficient of roughly 2.3E−2 mSv/MBq [23–25]. These results were summarised in the Supplementary Table 9. The authors believe that this difference is due to the different biodistribution of this radiopeptide. The previous studies calculated the absorbed dose in organs based on the biodistribution data of [^68^Ga]Ga-DOTATATE measured in patients who received this radiopeptide. In this work, the [^68^Ga]Ga-DOTATATE residence times were scaled from the ones obtained from [^177^Lu]Lu-DOTATATE [17]. This allowed the comparisons done in this study to be solely based on the physical properties of the different radionuclides evaluated and further reduce uncertainties associated with pharmacokinetics [27]. Nevertheless, these residence times scaling is likely to explain the differences observed in effective dose. Recently, Wong et al. [28] investigated the differences in tumour-to-normal organ standard uptake value (SUV) ratios measured with [^68^Ga]Ga-DOTATATE compared with [^177^Lu]Lu-DOTATATE in patients with NET. They reported that there is evidence of kinetic differences in DOTATATE uptake and internalization [28].

Other publications have identified the potential of using scandium radioisotopes for radiotheranostic application [29, 30]. Benabdallah et al. [30] have published preclinical and dosimetric estimation models for the scandium-44 in different scenarios, where they tried to observe and analyse the excretion of this radioisotope and the radiation impact in research and clinical use. Their dosimetry shows that the effective dose extrapolated from mice data to human, for male and female adults, were 0.146 mSv/MBq and 0.179 mSv/MBq, respectively. Compared to our findings, their values presented relative difference (Δ%) of 89% higher for both results. Moreover, they pointed out the significant absorbed dose in some organs for the scandium-44 due to the extended activity residence time of the radiopeptide in the body [30]. Analysing the AD/IA for the OARs, some their results are comparable to our calculations. Additionally, they reported [30], the heart, gall bladder, and stomach wall exhibited notably high absorbed doses. However, their biokinetic investigation was based on in vivo experiments with mice and different chemical ligand, which can explain the differences to our methodology. The chemical properties of the scandium radioisotopes allow their use for radiolabelling of other pharmaceuticals, for example to obtain [^44^Sc]Sc-PSMA-617 [31]. Despite the same radionuclide and different targeting agents, the biodistribution of the PSMA-617 presents the same regions of interest as the DOTATATE. Khawar et al. reported dosimetry for individual patients and the kidneys presented highest mean absorbed dose, while in our findings, the critical organ is the spleen followed by the kidneys [32].

Nevertheless, Singh et al. [3] published an imaging study for scandium-44 and gallium-68 labelled with DOTATATE. For one of the imaging scenarios presented, it was pointed out that there was a 9-month delay in detecting lesion using [^68^Ga]Ga-DOTATATE, and 24 h after [^44^Sc]Sc-DOTATATE administration the lesions were detected [3]. The suitability of scandium-44 compared to gallium-68 may guarantee the quality of the imaging ensuring that the regions of interest would have time enough to be seen [31]. Meanwhile, the dosimetry shows that the use of scandium radioisotopes would result in a higher absorbed dose compared to the same tumour-targeting agent labelled with the gallium, but reasonable differences with results found in literature for other radiopeptides with same aim.

Figure 4 shows the differences in effective dose between scandium-43, scandium-44 (including a 15% impurity of scandium-44m) and a mixture containing various ratios of both radioisotopes. The impurity of 15% was used to show the worst-case scenario, but as presented in the supplementary material, the presence of the scandium-44m impurity is almost negligible with a maximum of 8.1% increase in the effective dose. Regarding the differences found between the effective dose for scandium-43, scandium-44 and a possible mixture of scandium-43/scandium-44, the largest difference in effective dose was 6.8E−3 mSv/MBq for a sample containing only scandium-44 and 8.1E−3 mSv/MBq for a scandium-44 with 15% scandium-44m impurity. For current practices, these levels of variation in the absorbed dose are likely to be within dosimetry uncertainties of molecular radiotherapy dosimetry [33] and should not impact on the choice of radioisotope used. The choice will be made based on availability of the radionuclide and PET device technology, as some devices will not be able to quantitatively overcome the presence of the high gamma emission of scandium-44 and, thus, favour the use of scadium-43 [8, 9, 11]. Additionally, the use of a mixture of scandium-43/scandium-44 can overcome the cost associated with scandium-43 production by the use of same target material as for the production of scandium-44 irradiated at higher proton energies and benefit from additional information from the scandium-44 emissions for possible multi-isotope PET imaging [34]. In relation to overall effective dose, for an injected activity of 100 MBq (which was used previously [3]) a patient undergoing this examination would receive 0.92, 1.60, and 1.74 mSv, respectively for scandium-43, scandium-44, and scandium-44 and 44m mixture.

Some considerations about the source data used in dosimetry extrapolations performed in this work must also be discussed. The source region TIAC for lutetium-177 used to derive TIAC for the others PET tracers of concern in this study were reported by Marin et al. [17]. They applied bi-exponential fitting to derive TAC in the considered source regions. In their work, the acquired time-data points were obtained at 4, 24, and 168 h post therapeutic activity administration (p.a.). The relatively early first acquisition at 4 h p.a., with a second acquisition at 24 h p.a., possibly enabled a sufficiently reliable description of the first “fast” wash-out phase. In parenchymal organs, it is known that the TAC is mainly driven by a relatively “slow” radio-pharmacokinetics with effective half-lives, also reported by Marin et al., in the range of 50–80 h. For the case of [^177^Lu]Lu-DOTATATE, it has been reported that a loss of radio-pharmacokinetics information in the early hours after administration impacts the absorbed dose estimates in parenchymal organs of only a few percent (typically less than 5%) [22]. Possible TAC bias induced by the different PR-molecular probe conjugation, when compared to the [177Lu]Lu-DOTATATE from which the source TIAC used in this work were obtained, was not investigated in this work, but could possibly result in absorbed dose bias.

## Conclusion

The data of this study revealed a higher absorbed dose per injected activity of [^44^Sc]Sc-DOTATATE in organs at risk as compared to the dose delivered by [^68^Ga]Ga-DOTATATE, under the assumption that both radiopeptides would distribute equally. These findings can be attributed to the longer higher half-life of scandium radioisotopes as compared to that of gallium-68. As the calculated doses are within a reasonable order of magnitude of other clinically-used radionuclides, the suitability of scandium radionuclides to replace the gallium-68 for PET imaging would be feasible and potentially allow improved diagnostic interventions based on the possibility to image a patient at later time points after injection when the target-to-background contrast has increased.

### Supplementary Information

Additional file 1.

## Data Availability

The datasets used and/or analysed during the current study are available from the corresponding author on reasonable request.
