# Supplementary material for: Comparison of the dosimetry of scandium-43 and scandium-44 patient organ doses in relation to commonly used gallium-68 for imaging neuroendocrine tumours
Source: EJNMMI Phys. 2024 Jul 15;11:61. doi: 10.1186/s40658-024-00669-5 (PMC11247068; doi:10.1186/s40658-024-00669-5)
Supplement: Supplementary file 1 — Additional file 1. [file 40658_2024_669_MOESM1_ESM.docx]

Supplementary Materials

# Obtaining Absorbed dose per injected Activity

The Monte Carlo codes normalize their results by the number of histories simulated. In this case, the absorbed dose is normalized by the total emitted particles. For a given source-organ, the code will provide absorbed dose in the target-organs per particle emitted in the source-organ:

$$\frac{absorbed dose in target organ}{particle emitted in soure organ}$$

The ICRP DECDATA software provide the number of particles emitted per decay for a determined radioisotope:

$$\frac{particle emitted}{decay}$$

The time integrated activity coefficient (TIAC) related to a source organ, obtained from ICRP, calculated by the authors, or found in literature, will provide the time integrated activity (TIA) in the source-organ per injected activity, also called injected dose (ID) of the radiopeptide. The TIA provide the total number of decays in a source organ for a ID. Thus, you can write:

$$TIAC=\frac{TIA\left( source organ \right)}{ID}=\frac{Bq.s\left( source organ \right)}{ID}=\frac{decays in source organ}{ID}$$

For a given source-organ, the product of these three factors will return the absorbed dose in target organ per injected dose as we can see:

$$D\left( target\leftarrow source \right)=\frac{absorbeddose in target organ}{particle emitted in source organ}x\frac{particle emitted}{decay}x\frac{decays in sourceorgan}{ID}$$

Finally, the sum of contributions from all source-organs will provide the absorbed dose in each target-organ per injected dose (mGy/MBq).

# Summary of the decay data for the radionuclides evaluated in this study (ICRP 107)

Summary Decay Data Table for scandium-43

Half-Life: 3.891 h Specific Activity: 6.937E+20 Bq / kg

Mode: ECb + Source: ICRP-07.NDX

Frequency Energy Mean Energy

S Yi S Yi * Ei SYi * Ei / SYi

Radiation Number (/nt) (MeV/nt) (Mev)

Gamma rays 6 2.253E-01 8.436E-02 3.745E-01

X rays 17 4.562E-01 7.947E-05 1.742E-04

Annihilation quanta 1 1.760E+00 8.996E-01 5.110E-01

Beta + 2 8.802E-01 4.191E-01 4.761E-01

IC electrons 24 1.315E-04 4.855E-05 3.692E-01

Auger electrons 6 3.205E-01 3.561E-04 1.111E-03

Total Emitted Energy: 1.404E+00

Average energy of beta spectrum: 4.76E-01 MeV

End point energy of beta spectrum: 1.20E+00 MeV

Note: Yi = intensity of radiation i; Ei = energy of radiation i

Summary Decay Data Table for scandium -44

Half-Life: 3.97 h Specific Activity: 6.645E+20 Bq / kg

Mode: ECb + Source: ICRP-07.NDX

Frequency Energy Mean Energy

S Yi S Yi * Ei SYi * Ei / SYi

Radiation Number (/nt) (MeV/nt) (Mev)

Gamma rays 6 1.009E+00 1.173E+00 1.162E+00

X rays 17 2.151E-01 3.747E-05 1.742E-04

Annihilation quanta 1 1.887E+00 9.643E-01 5.110E-01

Beta + 1 9.435E-01 5.958E-01 6.315E-01

IC electrons 24 6.501E-05 7.510E-05 1.155E+00

Auger electrons 6 1.511E-01 1.679E-04 1.111E-03

Total Emitted Energy: 2.733E+00

Average energy of beta spectrum: 6.31E-01 MeV

End point energy of beta spectrum: 1.47E+00 MeV

Note: Yi = intensity of radiation i; Ei = energy of radiation i

Summary Decay Data Table for scandium -44m

Half-Life: 58.61 h Specific Activity: 4.501E+19 Bq / kg

Mode: ITEC Source: ICRP-07.NDX

Frequency Energy Mean Energy

S Yi S Yi * Ei SYi * Ei / SYi

Radiation Number (/nt) (MeV/nt) (Mev)

Gamma rays 4 9.028E-01 2.742E-01 3.038E-01

X rays 42 9.857E-01 9.812E-05 9.955E-05

IC electrons 16 1.212E-01 3.234E-02 2.668E-01

Auger electrons 13 8.300E-01 4.426E-04 5.332E-04

Total Emitted Energy: 3.071E-01

Note: Yi = intensity of radiation i; Ei = energy of radiation i

Summary Decay Data Table for lutetium-177

Half-Life: 6.647 d Specific Activity: 4.108E+18 Bq / kg

Mode: b - Source: ICRP-07.NDX

Frequency Energy Mean Energy

S Yi S Yi * Ei SYi * Ei / SYi

Radiation Number (/nt) (MeV/nt) (Mev)

Gamma rays 6 1.803E-01 3.156E-02 1.750E-01

X rays 60 1.374E+00 3.540E-03 2.576E-03

Beta - 4 1.000E+00 1.333E-01 1.333E-01

IC electrons 36 1.548E-01 1.352E-02 8.737E-02

Auger electrons 15 1.117E+00 1.132E-03 1.014E-03

Total Emitted Energy: 1.830E-01

Average energy of beta spectrum: 1.33E-01 MeV

End point energy of beta spectrum: 4.98E-01 MeV

Note: Yi = intensity of radiation i; Ei = energy of radiation i

Summary Decay Data Table for gallium-68

Half-Life: 67.71 m Specific Activity: 1.513E+21 Bq / kg

Mode: ECb + Source: ICRP-07.NDX

Frequency Energy Mean Energy

S Yi S Yi * Ei SYi * Ei / SYi

Radiation Number (/nt) (MeV/nt) (Mev)

Gamma rays 13 3.590E-02 3.961E-02 1.103E+00

X rays 25 5.689E-01 4.073E-04 7.158E-04

Annihilation quanta 1 1.778E+00 9.087E-01 5.110E-01

Beta + 3 8.891E-01 7.374E-01 8.293E-01

IC electrons 82 9.324E-06 9.792E-06 1.050E+00

Auger electrons 9 4.108E-01 5.499E-04 1.339E-03

Total Emitted Energy: 1.687E+00

Average energy of beta spectrum: 8.30E-01 MeV

End point energy of beta spectrum: 1.90E+00 MeV

Note: Yi = intensity of radiation i; Ei = energy of radiation i

Supplementary Tables 1 to 8 are the completed results of calculated absorbed dose per injected activity (AD/IA) for the adult female (AF) and adult male (AM) phantoms and

radioisotopes, respectively for scandium-43, scandium-44, scandium-44m, and gallium-68.

| Supplementary Table 1: AF – scandium-43 |
| --- |
| 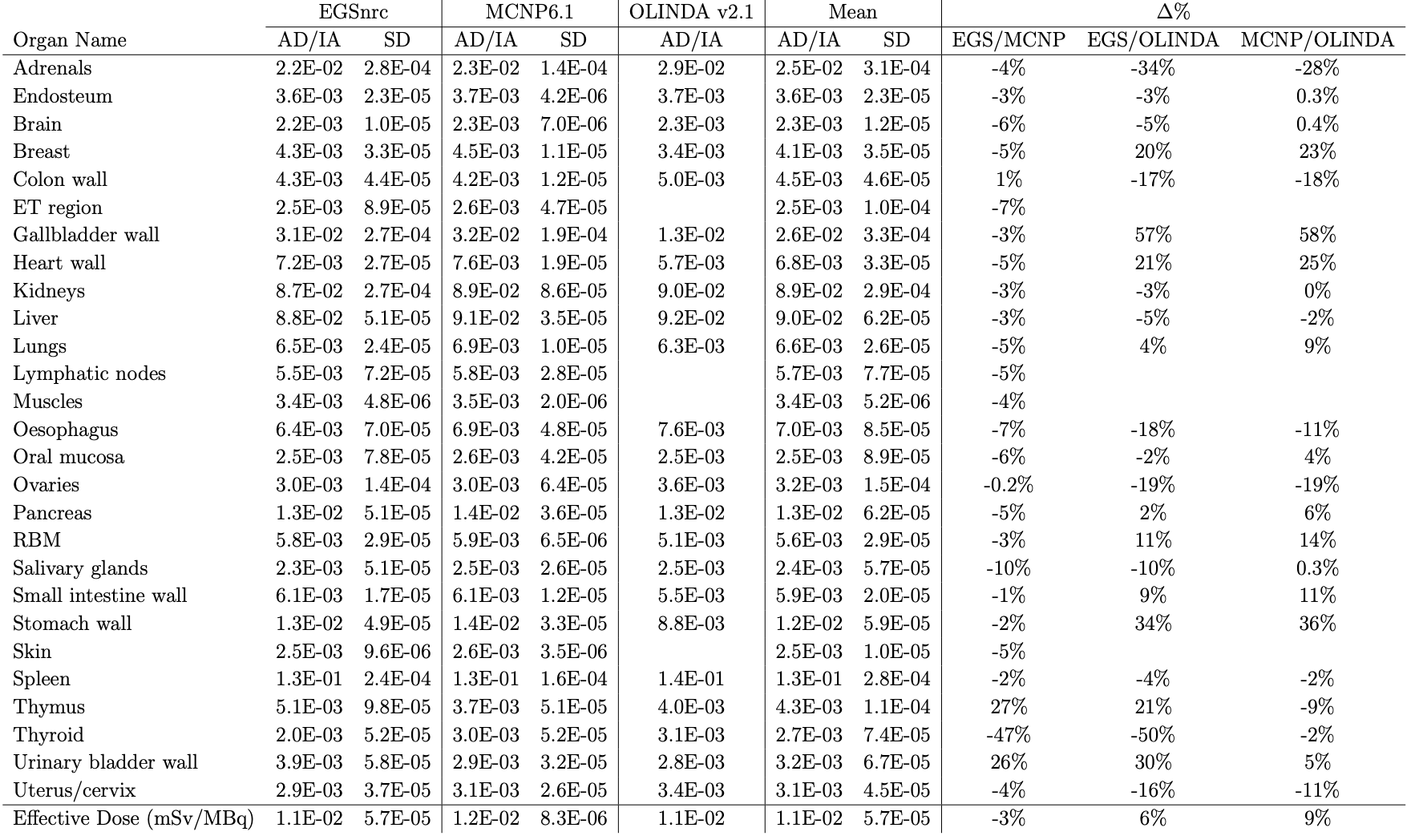 |
|  |
| Supplementary Table 2: AM – scandium-43 |
| 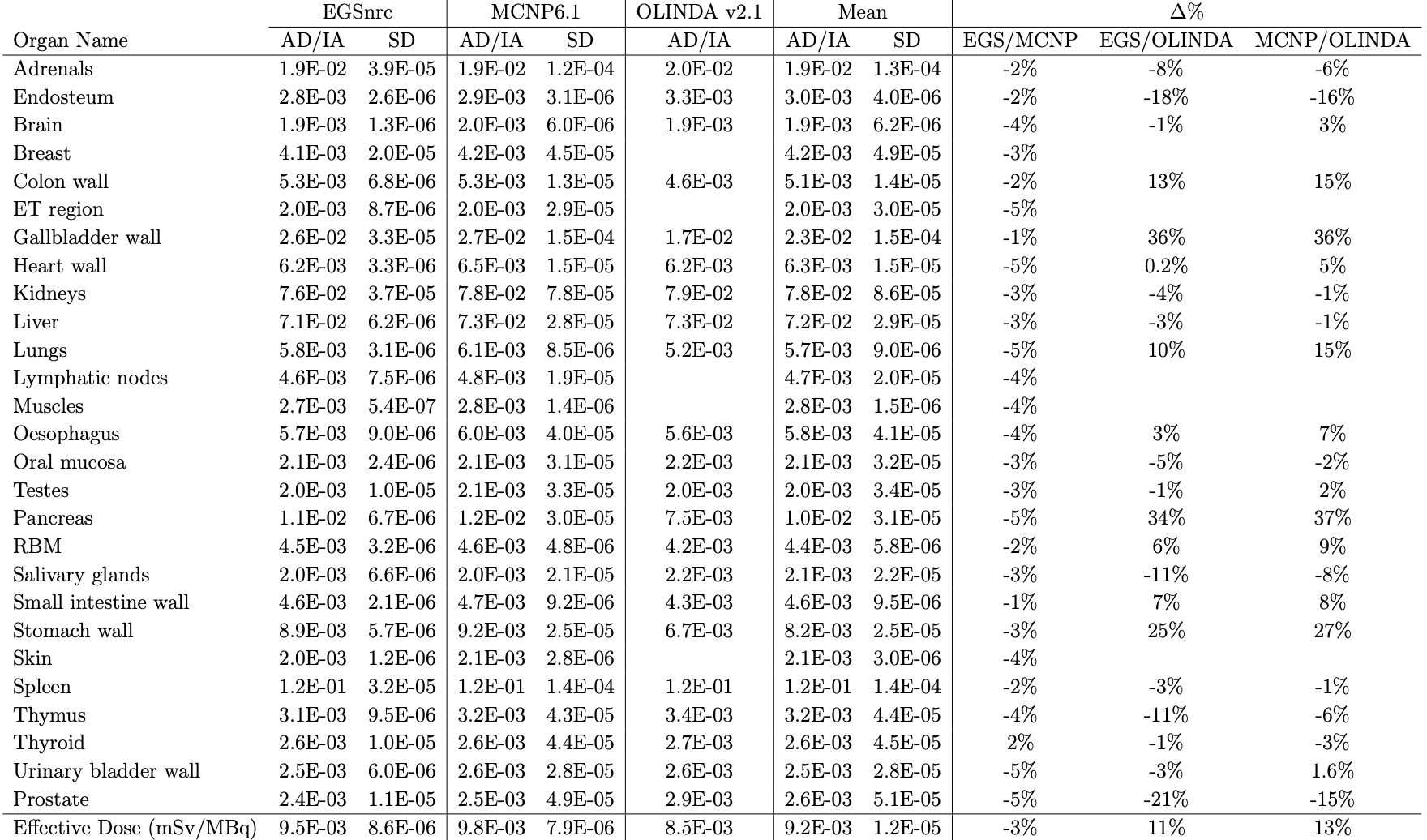 |
|  |
| Supplementary Table 3: AF – scandium-44 |
| 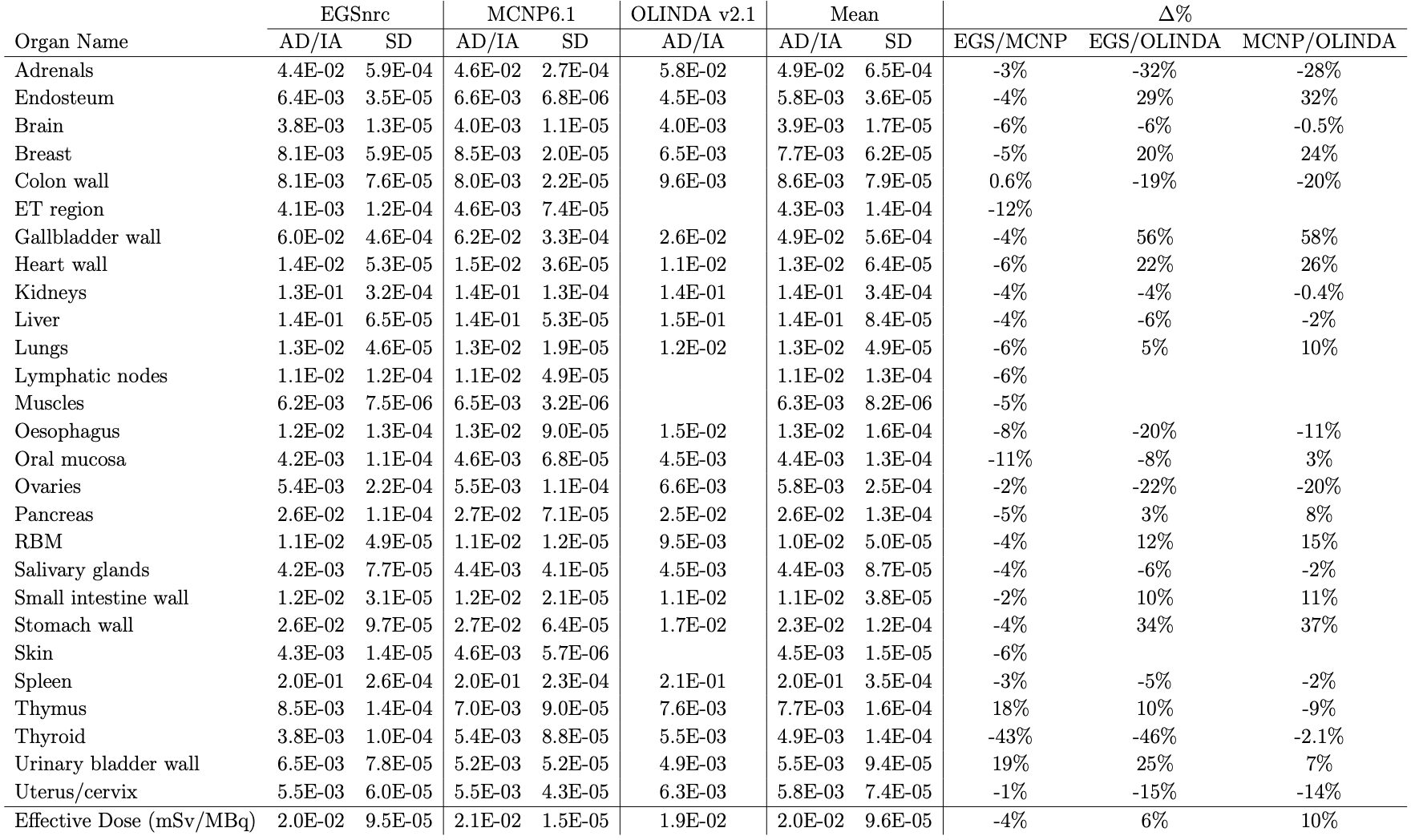 |
|  |
| Supplementary Table 4: AM – scandium-44 |
| 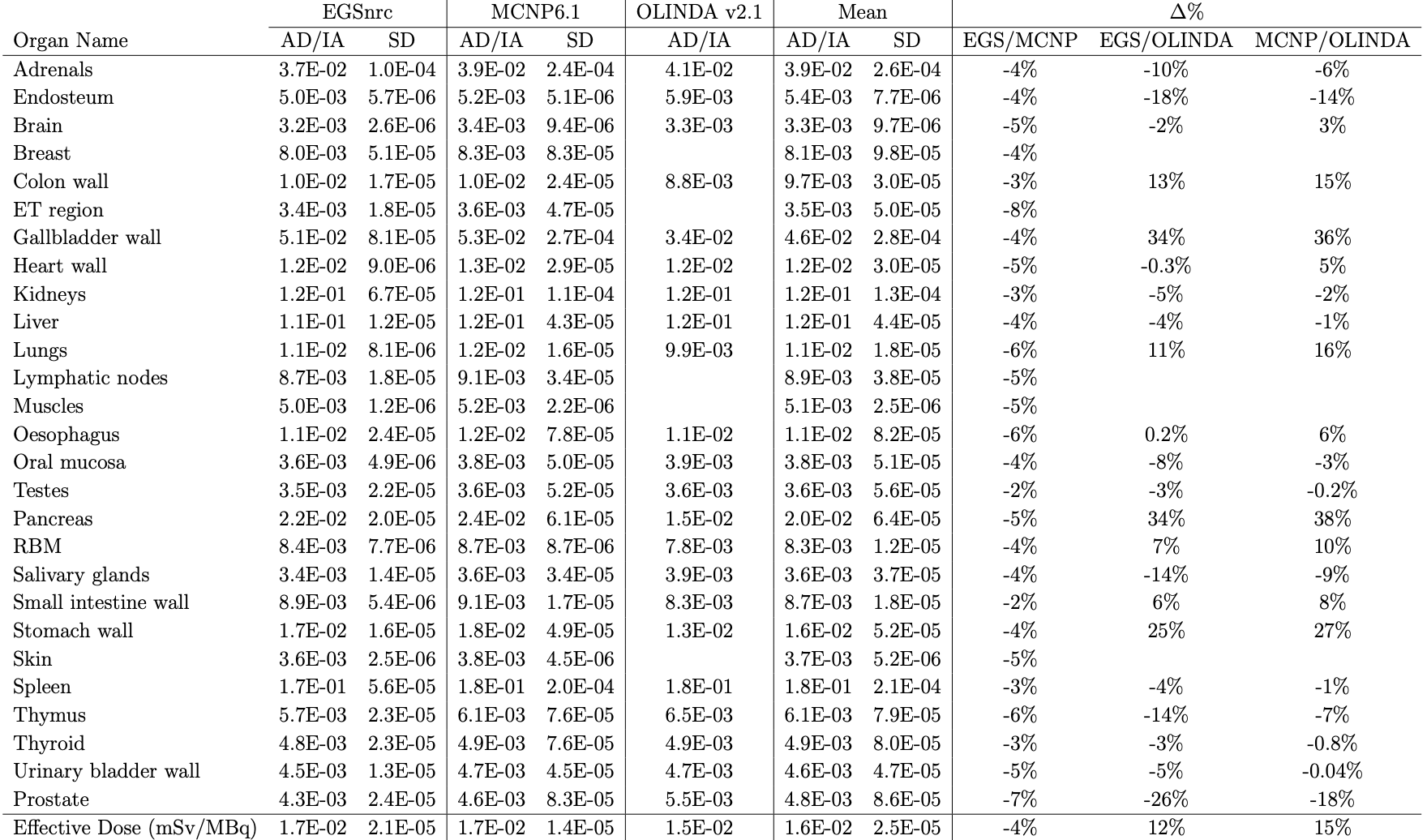 |
|  |
| Supplementary Table 5: AF – scandium-44m |
| 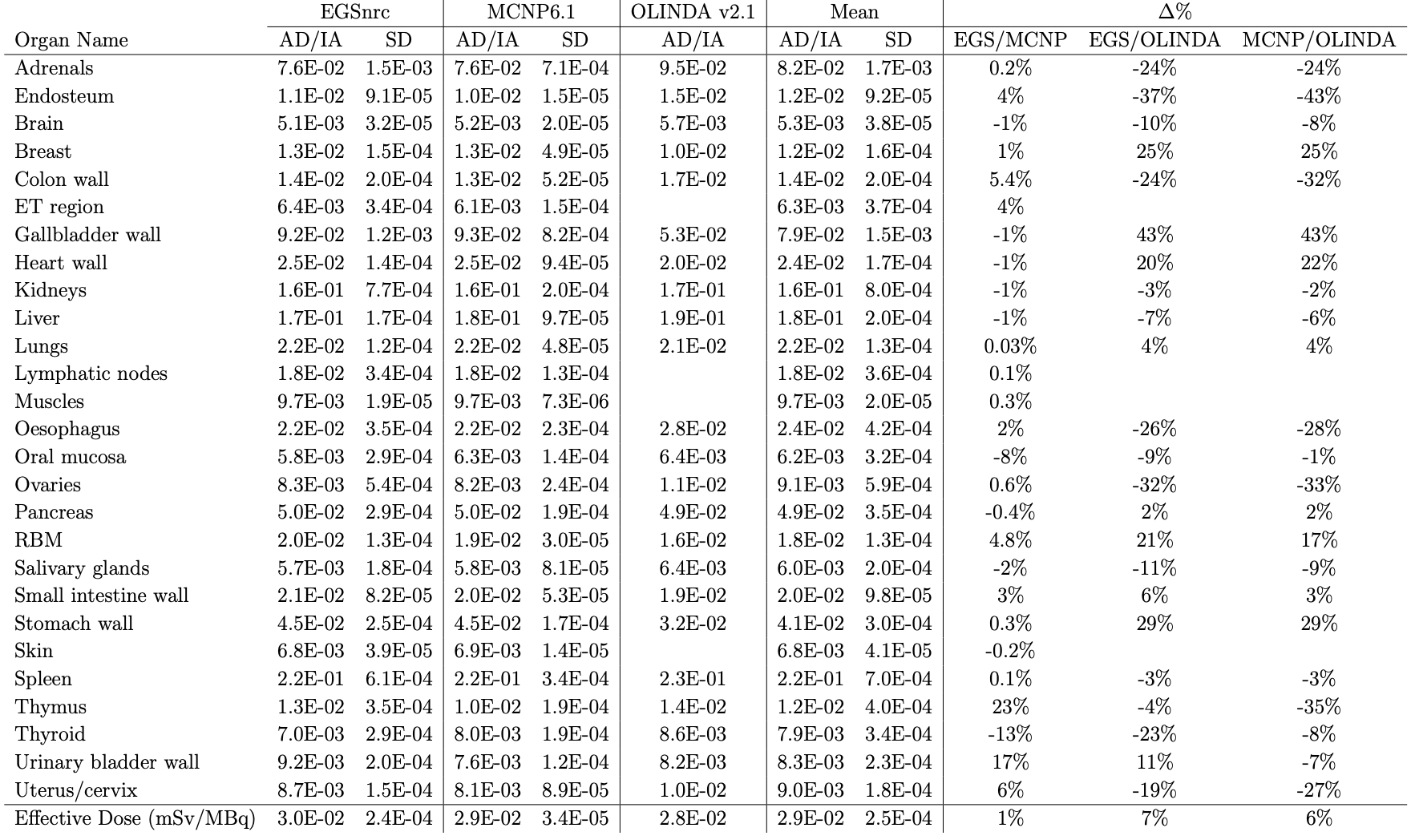 |
|  |
| Supplementary Table 6: AM – scandium-44m |
| 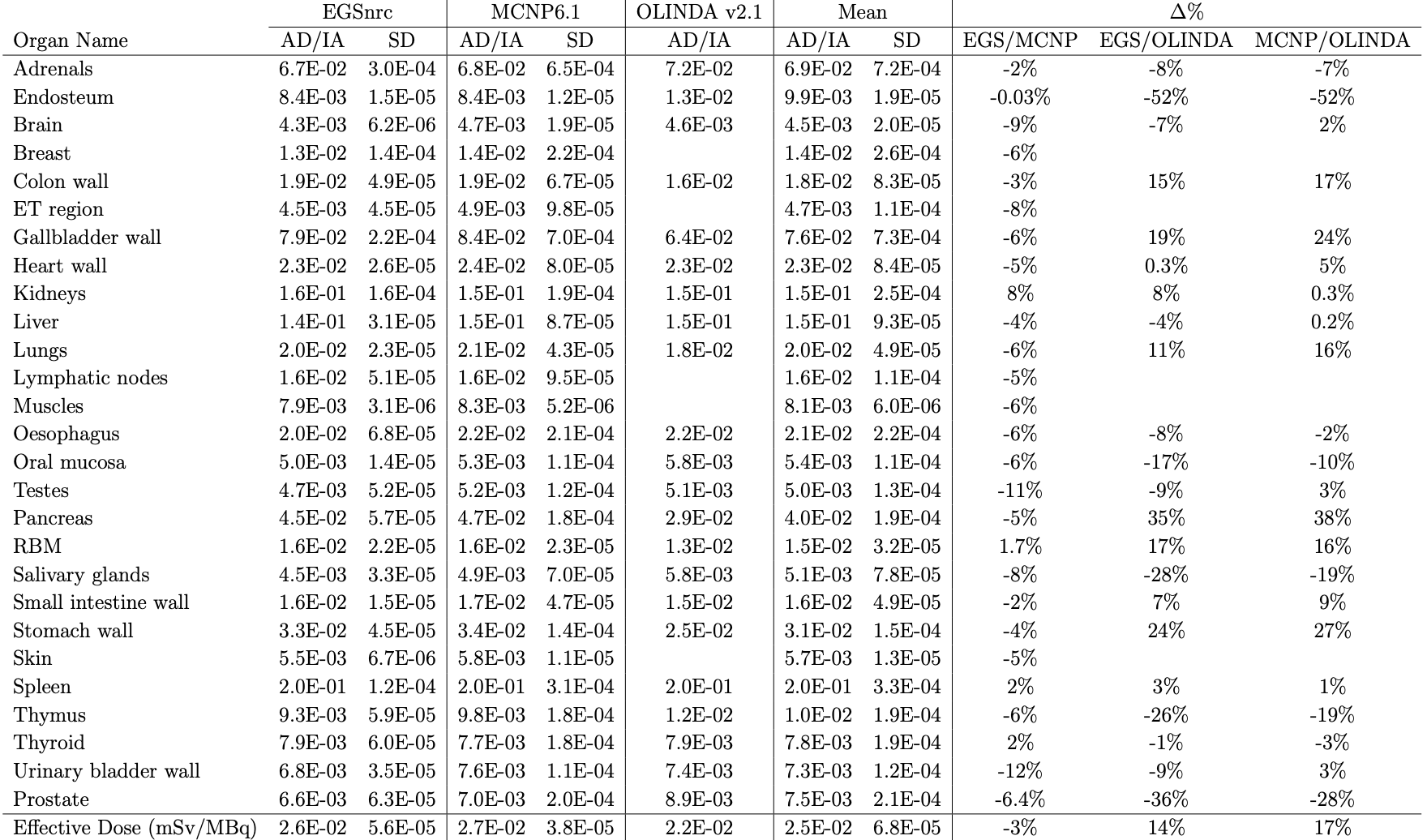 |
|  |
| Supplementary Table 7: AF – gallium-68 |
| 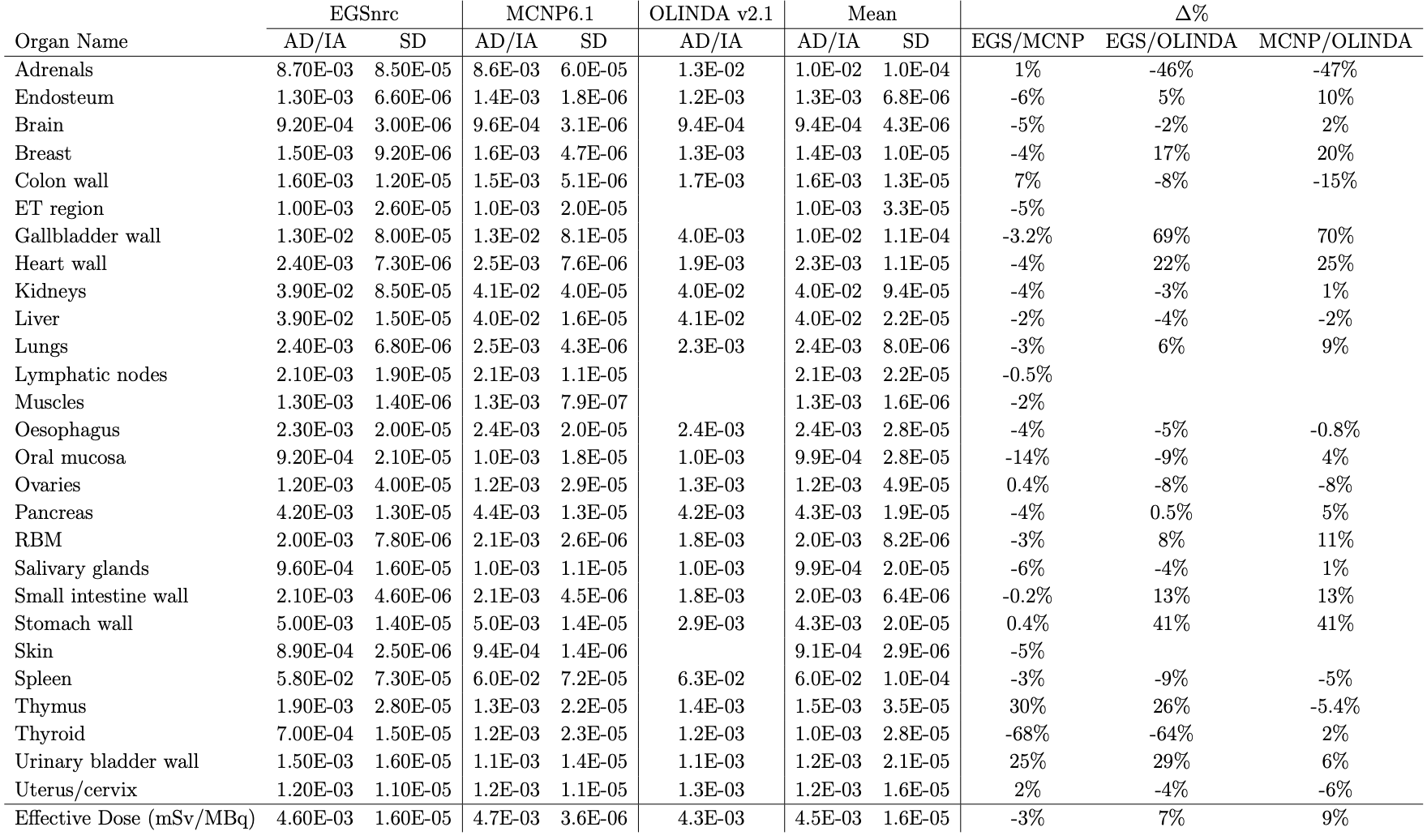 |
|  |
| Supplementary Table 8: AM – gallium-68 |
| 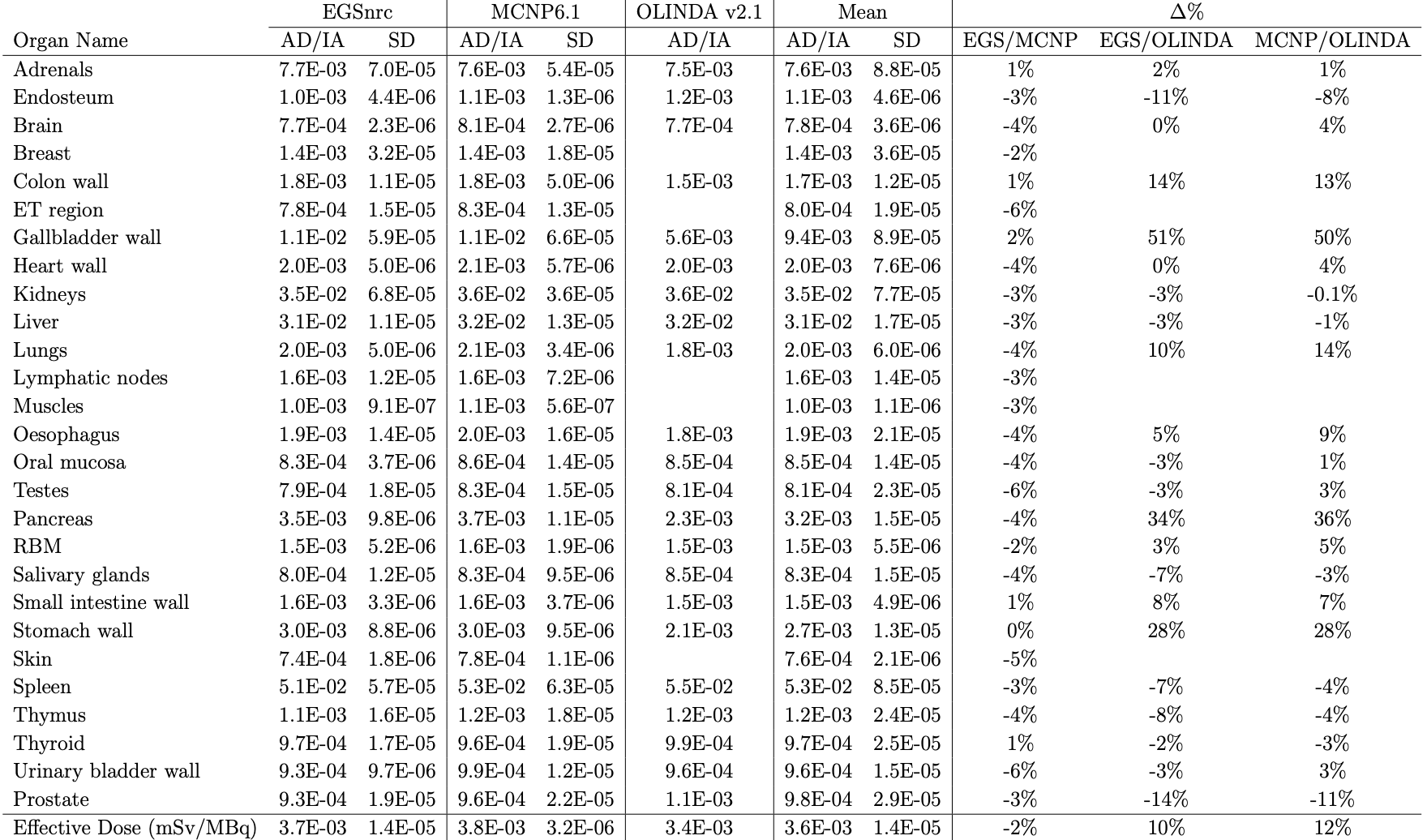 |

Impact of scandium-44 impurities in the effective dose.

For all evaluated concentrations of scandium-44m impurity the variation on the effective dose is almost negligible with the largest difference in the order of 5.3 e-3 mSv/MBq. Taking into account the effective dose contribution from the scandium-44, this would represent a maximum of 2.3% for 15% scandium-44m impurity in a scandium-44 sample. The supplementary figure 1 below shows the contributions for the different simulated impurities.


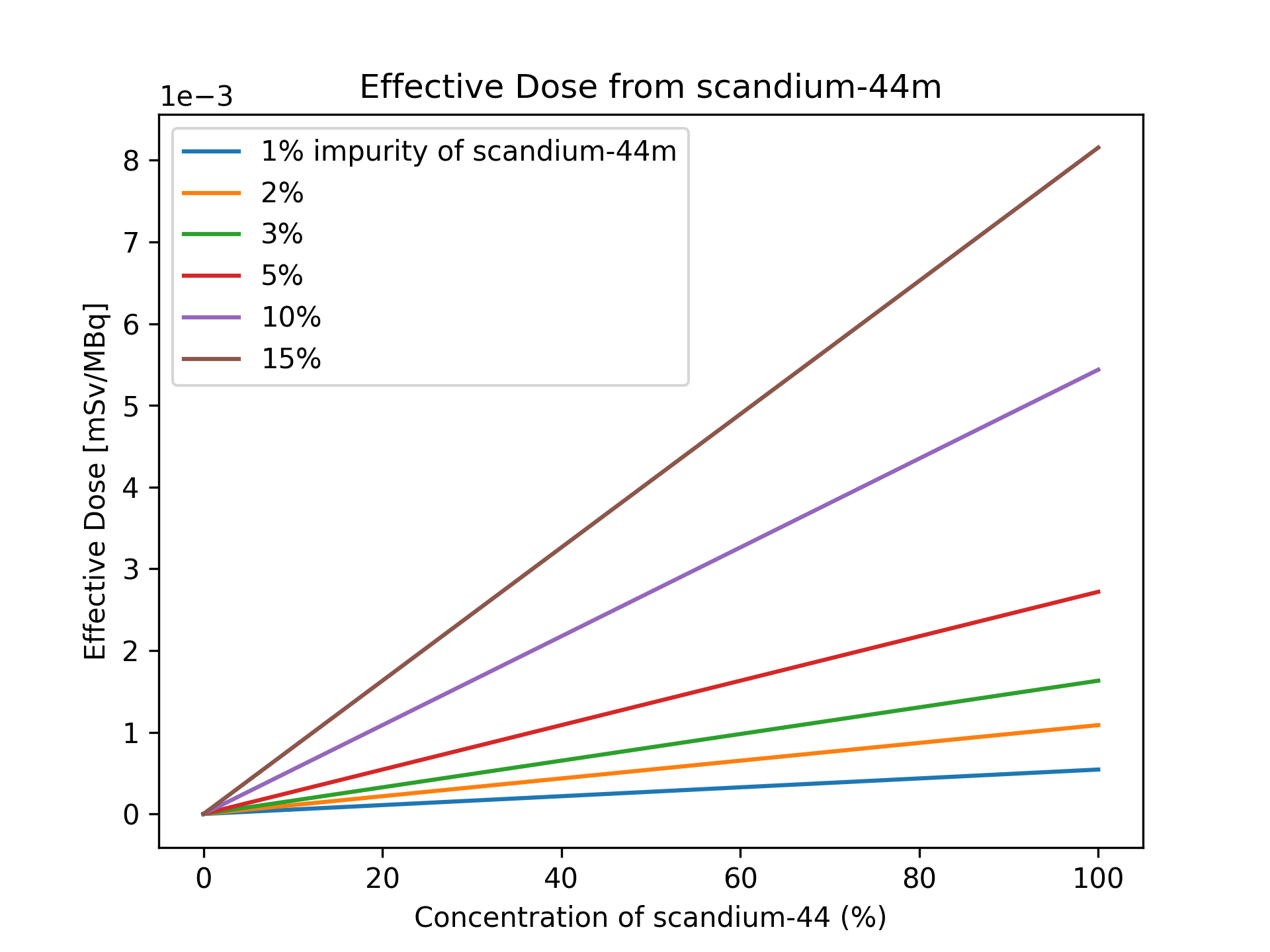


Supplementary Figure 1 – Introduced effective dose from the presence of different levels of scandium-44 impurity.

Supplementary table 9 presents the organ doses and effective dose obtained by this publication and found literature for gallium-68. These results were further presented in the discussion. The main difference for the found results was from the estimation on residence times for the organs which subsequently translated to differences in absorbed dose and effective dose.

Supplementary Table 9 – Calculated organ and effective doses by this study and the results found in the literature.

| 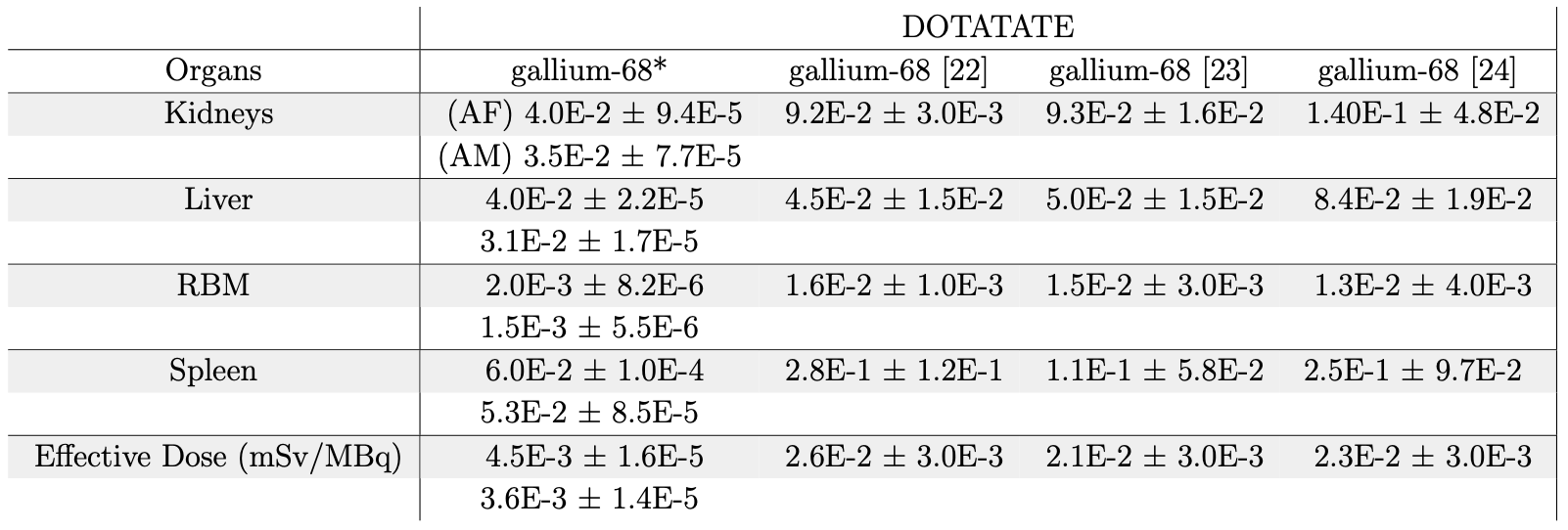 |
| --- |
| * Gallium-68 results obtained in this work. |
